# Value of diagnosing immunological phenomena in patients with suspected endocarditis

**DOI:** 10.1007/s15010-022-01954-0

**Published:** 2022-11-10

**Authors:** Thomas W. van der Vaart, Luca L. Heerschop, Berto J. Bouma, Wieke Freudenburg, Marc J. M. Bonten, Jan M. Prins, Jan T. M. van der Meer

**Affiliations:** 1 Department of Internal Medicine, Division of Infectious Diseases, Amsterdam UMC, University of Amsterdam, Meibergdreef 9, 1105 AZ Amsterdam, The Netherlands; 2 Julius Center for Health Sciences and Primary Care, University Medical Center Utrecht, Utrecht University, Heidelberglaan 100, 3584 CX Utrecht, The Netherlands; 3 Department of Cardiology, Amsterdam UMC, University of Amsterdam, Meibergdreef 9, 1105 AZ Amsterdam, The Netherlands; 4 Department of Medical Microbiology, Amsterdam UMC, University of Amsterdam, Meibergdreef 9, 1105 AZ Amsterdam, The Netherlands

**Keywords:** Endocarditis, Diagnosis, Duke Criteria, Roth’s spots, Immunological phenomena, Rheumatoid factor

## Abstract

**Purpose:**

Immunological phenomena are a minor criteria in the modified Duke Criteria for endocarditis. Given the changes in epidemiology and diagnostics, the added value of determining these phenomena in today’s patients with suspected endocarditis is unknown.

**Methods:**

In a retrospective cohort study of all patients with suspected endocarditis admitted to our hospital and discussed in our endocarditis team, we determined the proportion of patients classified as definite endocarditis because of either positive IgM rheumatoid factor (IgM RF), haematuria, or Roth’s spots on ophthalmology consultation. We also determined diagnostic accuracy of each of these immunological phenomena separately and combined.

**Results:**

Of 285 patients included, 138 (48%) had definite endocarditis and at least one immunological test was performed in 222 patients (78%). Elevated IgM RF was found in 22 of 126 patients tested (17%), haematuria in 78 of 196 tested (40%) and Roth’s spots in six of 120 tested (5%). Eighteen of 138 patients with definite IE (13%) were classified as such because of a positive IgM RF, haematuria or Roth’s spots. Haematuria had the highest sensitivity: 50.5% (95% CI 40.4–60.6) and Roth’s spots the highest specificity: 98.3% (95% CI 90.8–99.9). The diagnostic accuracy results were robust in a sensitivity analysis aimed at avoiding incorporation bias.

**Conclusion:**

Among patients with a clinical suspicion of endocarditis, recommended systematic testing for immunological phenomena helped classify more patients as definite IE and is useful to confirm the diagnosis of endocarditis.

**Supplementary Information:**

The online version contains supplementary material available at 10.1007/s15010-022-01954-0.

## Introduction

Infective endocarditis (IE) is a relatively rare but potentially fatal disease which can present with a myriad of signs and symptoms [1]. Because of its varying manifestations, the diagnosis of endocarditis can be challenging. Case definitions help to standardize clinical diagnosis and scientific reporting. Currently, the modified Duke Criteria are the reference standard for diagnosis [2–4]. Blood cultures and cardiac imaging are the cornerstones of the modified Duke Criteria, but not all patients with endocarditis meet these so-called major criteria. In these patients less specific signs and symptoms, minor criteria, may help to make the diagnosis. Among these are the immunological phenomena: Osler nodes, Roth’s spots, an elevated serum level of rheumatoid factor (IgM RF) and signs of glomerulonephritis. These immunological phenomena were common in earlier cohorts of patients with endocarditis and provide circumstantial evidence for the diagnosis of IE. Since the development of the Duke Criteria, the epidemiology of IE has changed, with higher prevalence of prosthetic cardiac material and electronic devices, an ageing patient population and increasing predominance of staphylococcal endocarditis [5, 6]. Also, diagnostic imaging for endocarditis has evolved, with 18-FDG PET/CT and cardiac CT added to the diagnostic workflow [7, 8]. With these changes in epidemiology and diagnostics, the question arises whether determining presence of immunological phenomena is still relevant for diagnosing IE.

In the Amsterdam University Medical Center (Amsterdam UMC), measuring IgM RF, urine sediment examination and ophthalmology consultation are recommended for all patients with suspected IE. The urine sediment is used to detect haematuria as a marker for glomerulonephritis [5], ophthalmology consultation to detect Roth’s spots. These investigations require additional laboratory testing and an ophthalmology consultation and thus incur additional healthcare costs. With improved echocardiography and imaging and more advanced microbiological methods, the added value of determining the presence of these immunological phenomena may have decreased. In this study, we determined their diagnostic value in a recent cohort of patients suspected of endocarditis.

## Methods

### Study objectives

In this study, we determined the proportion of patients with suspected endocarditis that were classified as ‘definite’ because of testing positive for IgM RF, haematuria, or Roth’s spots. Second, we determined the diagnostic accuracy of these immunological phenomena.

### Study design & setting

This study is an analysis of a prospective registry of patients suspected of endocarditis and discussed in the endocarditis team of the Amsterdam University Medical Centres, location AMC (AMC). The registry contains a cohort of patients directly admitted to the AMC, transferred patients and patients never admitted to the AMC but discussed in the endocarditis team for academic expertise. This project was exempt from Institutional Review Board (IRB) approval as it involved a retrospective analysis of routinely collected information (local IRB approval code: W21.157). This study is reported using the STROBE guidelines for reporting of observational studies [9].

### Participants

We included all consecutive adult patients discussed in the team who were directly admitted to the AMC between October 2016 and March 2021. Patients who were transferred from other hospitals or never admitted to the AMC were excluded because information on presence or absence of immunological phenomena was often missing in referral letters. Follow-up for this study was up to 90 days after admission and was conducted through chart review. Because the primary objective was to estimate the proportion of patients classified as definite endocarditis according to the ESC version of the modified Duke Criteria, we included all patients with suspected endocarditis, regardless of whether they were tested for immunological phenomena or not.

### Variables

For patients admitted to the AMC, the endocarditis team routinely recommends a diagnostic work-up with echocardiography (preferably transoesophageal echocardiography), blood cultures, serum IgM RF, urine sediment and an ophthalmology consultation, for all patients with suspected endocarditis. The treating physician decides whether immunological tests are performed.

The immunological phenomena were defined as a positive IgM RF as measured in serum (upper limit of normal range: 5 kIU/L), haematuria (> 17 erythrocytes/µL) or Roth’s spots seen by ophthalmology consultation. Dysmorphic erythrocytes or red blood cell casts were not required to meet the minor criterion of haematuria. Throughout this manuscript we will refer to these tests as immunological tests and the results as IgM RF, haematuria and Roth’s spots.

Osler nodes were not included in our research question, as Osler nodes, while also an immunological phenomenon, do not require further consultation or laboratory testing. We worked on this sentence before submission because indeed it can be hard to follow, especially if not familiar with the subject matter. In the end, this was the least confusing way of describing this process.

The primary outcome of the study was the proportion of patients that met the criteria of definite endocarditis only because one or more of the immunological phenomena was positive.

We also determined the diagnostic accuracy (sensitivity, specificity, positive and negative predictive values) of the immunological phenomena separate and combined for definite endocarditis as defined by the ESC-modified Duke Criteria [3].

A predisposing cardiac condition was defined as native valve disease with medium or high risk of endocarditis as defined previously [10], or presence of a prosthetic valve or cardiac implantable electronic device.

All data were collected from the electronic health record by the research physician or trained medical students supervised by the research physician.

### Statistical methods

Continuous data were reported as mean + standard deviation (sd) or median + interquartile range, based on visual examination for skewness. We examined differences between categorical variables using the Fisher-exact test. We performed a complete case analysis for each immunological test. Missing data were not imputed. The proportion of patients categorised as definite IE based on one or more positive immunological phenomena was reported with 95% binomial confidence intervals. The diagnostic accuracy (sensitivity, specificity, positive predictive value (PPV) and negative predictive value (NPV)), was calculated for all three index tests separately and combined, again with 95% binomial confidence intervals.

### Bias and sensitivity analyses

Since the modified Duke Criteria partially rely on the immunological phenomena to complete the diagnosis, incorporation bias may affect the evaluation of the contribution of immunological phenomena. To mitigate this, we performed a sensitivity analysis using only patients with pathology or surgery proven definite IE or definite IE based on having two major criteria, and patients with rejected IE. This removes all possible IE patients and all patients with a definite IE based on one major and three or more minor criteria or five minor criteria. In this sensitivity analysis only the diagnostic accuracy of the immunological tests are described, as the primary outcome (proportion of definite IE classified with use of the immunological phenomena) is not applicable to this subset.

All data were analysed using R version 4.1.2 (R: A language and environment for statistical computing. R Foundation for Statistical Computing, Vienna, Austria. https://www.R-project.org/).

## Results

### Patient characteristics and demographics

The database contained 597 patients discussed in the endocarditis team between 1 October 2016 and 1 March 2021. Of these, 226 were excluded because the primary admitting hospital was not the AMC and 86 were excluded because they were never transferred but discussed for academic expertise only. This resulted in 285 patients who were directly admitted and had suspected endocarditis. Of these 285, 138 (48%) had definite IE, 115 (40%) were classified as possible IE and IE was rejected in 32 (11%). Native valve endocarditis was present in 72/138 patients (52%), prosthetic valve endocarditis in 51 (37%) and cardiac implantable electronic device endocarditis in 15 (11%). Important demographic and clinical information is presented in Table 1. Five patients with rejected endocarditis underwent cardiac surgery, four of whom underwent surgery for valvular regurgitation which proved not to be endocarditis on macroscopic and pathological examination and one who had purulent pericarditis requiring surgery but no evidence of endocarditis.

**Table 1:** Demographic and clinical characteristics

	All patients	Definite IE	Possible IE	Rejected IE
*n*	285	138	115	32
Demographics				
Age (median [IQR])	63 [49–74]	64 [48–75]	63 [52–74]	63 [52–71]
Male	69 (198/285)	75 (104/138)	65 (75/115)	59 (19/32)
Ischaemic heart disease	16 (47/285)	17 (24/138)	15 (17/115)	19 (6/32)
Heart failure	52 (149/285)	59 (82/138)	43 (50/115)	53 (17/32)
Cyanotic heart disease	7 (20/285)	9 (12/138)	4 (5/115)	9 (3/32)
Previous endocarditis	8 (23/285)	7 (10/138)	7 (8/115)	16 (5/32)
Diabetes	23 (66/285)	18 (25/138)	26 (30/115)	34 (11/32)
Charlson score (median [IQR])	2 [1–4]	2 [1–4]	3 [1–5]	3 [2–4]
Prosthetic valve	34 (96/285)	41 (57/138)	24 (28/115)	34 (11/32)
Cardiac electonic device				
CRT-D	2 (7/285)	1 (2/138)	4 (5/115)	0 (0/32)
ICD	5 (13/285)	7 (9/138)	3 (3/115)	3 (1/32)
Pacemaker	11 (31/285)	14 (19/138)	5 (6/115)	19 (6/32)
Clinical features				
Fever	66 (188/285)	78 (107/138)	62 (71/115)	31 (10/32)
Vascular phenomena^a^	35 (100/285)	41 (56/138)	35 (40/115)	13 (4/32)
Immunological phenomena	33 (93/285)	43 (60/138)	24 (28/115)	16 (5/32)
Osler nodes	1 (4/285)	3 (4/138)	0 (0/115)	0 (0/32)
IgM RF^b^	17 (22/126)	19 (13/67)	16 (8/51)	13 (1/8)
Haematuria^c^	40 (78/196)	50 (51/101)	28 (23/81)	29 (4/14)
Roth’s spots^d^	5 (6/120)	8 (5/62)	2 (1/50)	0 (0/8)
Imaging major criterion	49 (140/285)	85 (117/138)	17 (20/115)	9 (3/32)
Microbiology major criterion	65 (184/285)	83 (115/138)	60 (69/115)	0 (0/32)
Causative microorganism				
Streptococci	19 (54/285)	24 (33/138)	17 (20/115)	3 (1/32)
* S. aureus*	34 (97/285)	33 (46/138)	38 (44/115)	22 (7/32)
Coagulase negative staphylococci	10 (28/285)	13 (18/138)	7 (8/115)	6 (2/32)
Enterococci	7 (20/285)	10 (14/138)	5 (6/115)	0 (0/32)
* HACEK* group	2 (6/285)	4 (5/138)	1 (1/115)	0 (0/32)
Culture negative	16 (46/285)	4 (6/138)	18 (21/115)	59 (19/32)
Other	12 (34/285)	12 (16/138)	13 (15/115)	9 (3/32)
Clinical outcomes				
Underwent cardiac surgery	14 (40/285)	22 (31/138)	3 (4/115)	16 (5/32)
90-day mortality^e^	19 (52/270)	18 (23/131)	21 (23/107)	19 (6/32)
90-day relapse rate^f^	4 (9/236)	3 (4/131)	6 (5/90)	0 (0/15)

Numbers are % (*n*/*N*) unless otherwise indicated

*ICD* implantable cardioverter defibrillator, *CRT-D* cardiac resynchronization therapy defibrillator, *HACEK:* *Haemophilus*, *Aggregatibacter*, *Cardiobacterium*, *Kingella*, *Eikenella*

^a^As defined by the modified Duke criteria: septic embolism, Janeway lesions, septic pulmonary infarction, mycotic aneurysm, conjunctival haemorrhages

^b^Not performed in 159 patients

^c^Not performed in 89 patients

^d^Not performed in 165 patients

^e^Data missing in 15 patients

^f^Data missing in 49 patients

### Results of immunological tests

In 222 patients (78%) at least one immunological test was performed. An elevated IgM RF was found in 22 of 126 tested patients (17%), while haematuria was present in 78 of196 tested patients (40%). Two patients had dysmorphic erythrocytes or red cell casts, one of whom had definite endocarditis. Roth’s spots were seen in 6 of 120 patients (5%) who had an ophthalmology consultation. A detailed breakdown of performed tests and missing data is provided in Table 1. Immunological tests were performed in 112/138 patients with definite IE (81%), in 94/115 patients with possible IE (82%) and in 16/32 patients with rejected IE (50%).

A positive immunological test was common in *Staphylococcus aureus*, enterococcal, and *HACEK* infection: 38/77 (49%), 8/17 (47%) and 4/5 (80%) of tested patients, respectively. Streptococcal, coagulase negative staphylococcal and culture-negative IE were less likely to cause immunological phenomena: 14/47 (30%), 7/22 (32%) and 11/33 (33%) of tested patients. The differences between these groups were not statistically significant (*p* = 0.14 for the Fisher-exact test across all groups).

### Effect of immunological tests on Duke classification

Of 138 patients with definite IE according to the ESC-modified Duke Criteria, 18 (13.0%; 95% CI 7.9–19.8%) met this definition by testing positive on urine sediment or IgM RF or having Roth’s spots on ophthalmology consultation. The majority of these patients (*n* = 15) had haematuria, five patients had a positive IgM RF and two patients had Roth’s spots. Four patients had more than one immunological phenomenon: three patients had both haematuria and a positive IgM RF and one patient had haematuria and Roth’s spots. No patient had all three immunological phenomena.

Of the 18 patients classified as definite IE because of immunological tests, 12 (67%) had native valve endocarditis (NVE), five (28%) had prosthetic valve endocarditis (PVE) and one (6%) had device endocarditis. In eight (44%) of these patients, endocarditis was caused by *S. aureus*. Three patients (17%) met the major imaging criterion of the ESC-modified Duke Criteria, while 15 (83%) met the major microbiological criterion for IE. More detail on the reclassified patients is provided in Table 2. None of the patients reclassified due to haematuria had a positive urine culture at the time of collection of the urine sediment.

**Table 2:** Overview of patients reclassified due to immunological phenomena

Patient	Gender, age	Causative microorganism	Duration of bacteraemia	Number of positive blood cultures	Cardiac predisposition	Immunological phenomena	Metastatic and vascular signs of infection	Imaging	Major Duke criteria	Minor Duke criteria
1	Male, 74	*Staphylococcus aureus*	3 days	4	Pacemaker	Glomerulonephritis (haematuria, leukocyuria)	–	No evidence of IE on PET/CT or TTE, contraindication to TEE	Microbiological	Fever Predisposition Immunological
2	Male, 59	*Streptococcus sanguinis*	2 days	6	Bicupisd aortic valve, previous endocarditis	Glomerulonephritis (haematuria)	–	On TEE increased gradient over native bicuspid aortic valve, valvular thickening but no vegetations	Microbiological	Fever Predisposition Immunological
3	Female, 47	*Staphylococcus aureus*	7 days	6	None	Glomerulonephritis (haematuria)	Septic pulmonary emboli	On TEE subtle valve thickening of AMVL, no visible vegetations. PET/CT pulmonary emboli and septic emboli in the lower spine	Microbiological	Fever Vascular Immunological
4	Female, 70	*Staphylococcus aureus*	2 days	2	Severe MR and TR	IgM RF	–	TTE: increased valvular thickening of the native aortic valve	Microbiological	Fever Predisposition Immunological
5	Male, 60	Culture negative	NA	0	None	Glomerulonephritis (haematuria) IgM RF	Stroke	TEE: 12 mm vegetation on mitral valve	Imaging	Fever Vascular phenomena Immunological
6	Female, 86	*Streptococcus pneumoniae*	1 day (3 cultures over 12 h)	3	Moderate TR, mild MR	Glomerulonephritis (haematuria)	Septic arthritis of shoulder and knee	TTE: thickening of AoV, increased compared to previous TTE Died before TEE possible	Microbiological	Fever Predisposition Immunological
7	Male, 84	*Enterococcus faecium*	1 day (5 cultures over 14 h)	5	TAVI, pacemaker	Glomerulonephritis (hematuria)	–	No clear evidence on TEE/TTE/PET-CT or cardiac CT	Microbiological	Fever Predisposition Immunological
8	Male, 75	*Streptococcus sanguinis*	2 days	5	TAVI	Glomerulonephritis (haematuria)	–	No clear evidence on TTE/PET-CT or cardiac CT. TEE refused by patient	Microbiological	Fever Predisposition Immunological
9	Female, 75	*Staphylococcus epidermidis*	2 days	4	Mechanic mitral and aortic valves	IgM RF	–	TEE: slightly increased paravalvular leakage mitral valve PET/CT: no evidence of IE	Microbiological	Fever Predisposition Immunological
10	Female, 49	*Staphylococcus lugdunensis*	2 days	3	TAVI	Glomerulonephritis (haematuria, leukocyturia)	–	TEE and PET/CT no clear evidence of IE	Microbiological	Fever Predisposition Immunological
11	Male, 72	Culture negative	NA	0	None	Glomerulonephritis (haematuria) IgM RF	Distal septic emboli, Janeway lesions	TEE vegetation AoV (12 mm)	Imaging	Fever Vascular Immunological phenomena
12	Male, 75	*Escherichia coli*	1 day (2 blood cultures)	2	None	Glomerulonephritis (haematuria)	–	TEE and TTE: vegetation AMVL (8 mm)	Imaging	Fever Immunological Microbiological
13	Male, 70	*Staphylococcus aureus*	2 days	4	Bioprosthetic aortic valve	Glomerulonephritis (haematuria)	–	TEE no vegetations	Microbiological	Fever Predisposition Immunological
14	Male, 48	*Staphylococcus aureus*	2 days	7	None	Glomerulonephritis (haematuria, leukocyturia)	Janeway laesions	TTE and TEE no vegetations	Microbiological	Fever Vascular Immunological
15	Female, 65	*Staphylococcus haemolyticus*	6 days	8	Moderate TR and MR	Glomerulonephritis (haematuria, leukocyturia) Roth’s spots	–	TTE and PET/CT no endocarditis or other focus	Microbiological	Fever Predisposition Immunological
16	Male, 81	*Staphylococcus aureus*	1 day (2 blood cultures)	2	Moderate MR	Glomerulonephritis (haematuria) IgM RF	Splinter haemorrhages Vertebral osteomyelitis	TTE and TEE no clear IE, but thickening of MV and increase in MR after repeat TTE	Microbiological	Fever Predisposition Immunological
17	Male, 86	*Staphylococcus aureus*	7 days	8	Moderate TR, mild MR	Glomerulonephritis (haematuria, leukocyturia)	Vertebral osteomyelitis, splinter haemorrhages	TTE and TEE no clear IE	Microbiological	Fever Predisposition Immunological
18	Male, 78	*Staphylococcus aureus*	2 days	4	Moderate MR	Roth’s Spots	Infected total hip prosthesis	TTE and TEE no clear IE	Microbiological	Fever Predisposition Immunological

*IE* infective endocarditis, *AMVL* anterior mitral valve leaflet, *AoV* aortic valve, *MR* mitral valve regurgitation, *TR* tricuspid valve regurgitation, *TTE* transthoracic echocardiogram, *TEE* transoesophageal echocardiogram, *TAVI* Transcatheter Aortic Valve Implantation, *PET/CT* 18-FDG positron emission tomography/computed tomography

### Diagnostic accuracy

The diagnostic accuracy of each immunological phenomenon for the diagnosis definite IE is listed in Table 3, with two-by-two tables provided as supplemental tables S1–S3. Only patients who underwent the test were used in this analysis; patients with missing tests results were not analysed. Presence of Roth’s spots had the highest specificity: 98.3% (95% CI 90.8–99.9), while sensitivity was highest for haematuria: 50.5% (95% CI 40.4–60.6) None of the immunological phenomena had a sensitivity above 51%, and the combination of the three tests improved sensitivity only slightly.

**Table 3:** Diagnostic accuracy of immunological tests

Phenomena	Sensitivity (%; 95% CI) #	Specificity (%; 95% CI)	Negative predictive value (%; 95% CI)	Positive predictive value (%; 95% CI)
Haematuria (*n* = 196)^a^	50.5 (40.4–60.6)	71.6 (61.4–80.4)	57.6 (48.2–66.7)	65.4 (53.8–75.8)
Positive IgM RF (*n* = 126)^a^	19.4 (10.8–30.9)	84.7 (73.0–92.8)	48.1 (38.2–58.1)	59.1 (36.4–79.3)
Roth’s spots (*n* = 120)^a^	8.1 (2.7–17.8)	98.3 (90.8–99.9)	50.0 (40.5–59.5)	83.3 (35.9–99.6)
Any of the above three (*n* = 222)^a^	51.8 (42.1–62.1)	70.0 (60.5–78.4)	58.8 (49.8–67.3)	63.4 (53.0–73.6)

^a^Test performed and test result known

### Sensitivity analysis

To mitigate incorporation bias we performed a sensitivity analysis. In this analysis patients who had definite endocarditis based on two major criteria or meeting the pathological definition of IE (*n* = 87) were compared to patients with rejected IE (*n* = 32). The point estimates for sensitivity and specificity were comparable to those in the main analysis, but the predictive values skewed towards more extreme as is to be expected in this more selected population with a different disease prevalence (Table 4).

**Table 4:** Diagnostic accuracy of immunological tests (sensitivity analysis)

Test	Sensitivity (%; 95% CI) #	Specificity (%; 95% CI)	Negative predictive value (%; 95% CI)	Positive predictive value (%; 95% CI)
Haematuria (*n* = 71)^a^	49.1 (35.6–62.7)	71.4 (41.9–91.6)	25.6 (13.0–42.1)	87.5 (71.0–96.5)
Positive IgM RF (*n* = 46)^a^	15.8 (6.0–31.3)	87.5 (47.3–99.7)	17.9 (7.5–33.5)	85.7 (42.1–99.6)
Roth’s spots (*n* = 47)^a^	5.1 (0.6–17.3)	100 (63.1–100)	17.8 (8.0–32.1)	100 (15.8–100)
Any of the above three (*n* = 81)^a^	46.2 (33.7–59.0)	68.8 (41.3–89.0)	23.9 (12.6–38.8)	85.7 (69.7–95.2)

^a^Test performed and test result known

## Discussion

In this retrospective cohort study, we found that 18 (13%) of 138 patients with definite IE were classified as definite because of haematuria, a positive IgM RF or presence of Roth’s spots. We found that haematuria was the most sensitive of the immunological phenomena, while IgM RF and Roth’s spots were less sensitive but more specific. All these findings were robust in the sensitivity analysis.

### Study in context

Among patients tested the prevalence of haematuria (40%), IgM RF (17%) and Roth’s spots (5%) was about twice as high in previous cohort studies [5, 8, 11–13]. These studies however often did not report how patients not tested were evaluated [5, 8, 13] or did not report how the immunological phenomena impacted the diagnostic classification [11, 12]. The prevalence of an elevated IgM RF as found in our study is in line with the prevalence of 24% for rheumatoid factor reported in the older IE cohort by Von Reyn from 1981, who was the first to suggest immunological phenomena as a marker for IE, though Von Reyn classified them as vascular phenomena [14]. The same applies for our prevalence of 40% for haematuria, which is in line with prevalences of around 50% found in studies from this earlier time period [15, 16]. We hypothesize that the lower rates found in other recent studies reflect a lower proportion of patients with endocarditis tested, or that only the patients with a lower probability of endocarditis and resulting immunological phenomena were tested. It is also conceivable that other studies assumed that patients not tested had a negative test result, which would also result in a falsely low prevalence.

The diagnostic accuracy of the immunological phenomena appears limited, with most phenomena having NPVs of around 50% and PPVs of around 60%. The minor criteria of the Duke Criteria, however, are by definition clinical signs and symptoms with limited diagnostic value on their own. The Duke Criteria work because they combine these imperfect tests to form a final conclusion that has acceptable sensitivity and very good specificity. As such, systematic testing will lead to more patients with IE being able to be classified as definite IE.

### Strengths and limitations

We present a large, recent and well-documented cohort of patients with both proven and suspected IE where the immunological phenomena were frequently tested according to a standard protocol. Adherence to protocol was reasonable, as 78% of patients received at least one immunological test. However, despite the fact that a large proportion of patients was tested for immunological phenomena, the testing was not performed in all patients and this may have been influenced by confounding by indication: it is conceivable that the performance of immunological tests was related to the estimated likelihood of endocarditis. Conversely, immunological tests may have been withheld in patients with imaging-proven endocarditis and may be more likely performed in patients with (initially) negative or inconclusive imaging. Evaluation of the diagnostic accuracy of immunological phenomena using the modified Duke Criteria as a reference standard results by definition in incorporation bias, as the immunological phenomena are part of the criteria. In the sensitivity analysis, which avoids incorporation bias, the point estimates for sensitivity and specificity were comparable to those in the main analysis. Haematuria alone is only a marker of possible glomerulonephritis, and though this has been used before as a marker for glomerulonephritis, it is possible that this non-specific marker could have led to a number of patients wrongly classified as having glomerulonephritis, which could theoretically result in to decreased specificity of the Duke Criteria [5]. Last, we did not evaluate whether the reclassification due to the immunological phenomena led to changes in patient management. It is, however, important to realize that the Duke Criteria were mainly developed as a method for selecting patients for clinical research and should not replace clinical judgement [2–4, 17]. As such, it is important to realize that presence of one or more immunological phenomena may be helpful for clinicians to create greater certainty in the diagnosis of IE, but that absence of immunological phenomena should not be used to rule out endocarditis, and patiens with a high index of suspicion for IE should be treated at the clinicians discretion. 

### Conclusion

Recommended systematic testing for immunological phenomena in patients with suspected endocarditis leads to a small but relevant increase in patients classified as definite IE. Especially in patients with a high index of suspicion but lacking positive imaging or positive microbiology results, testing for immunological phenomena is useful to confirm the diagnosis of endocarditis.

## Supplementary Information

Below is the link to the electronic supplementary material.

Supplementary file1 (DOCX 16 KB)
