# Supplementary material for: Value of diagnosing immunological phenomena in patients with suspected endocarditis
Source: Infection. 2022 Nov 10;51(3):705–13. doi: 10.1007/s15010-022-01954-0 (PMC10205820; doi:10.1007/s15010-022-01954-0)
Supplement: Supplementary file 1 — Supplementary file1 (DOCX 16 KB) [file 15010_2022_1954_MOESM1_ESM.docx]

**Supplemental table S1: 2x2 table for haematuria and definite endocarditis**

|  | **Endocarditis** | **No endocarditis** | **Total** |
| --- | --- | --- | --- |
| **Haematuria** | 51 | 27 | 78 |
| **No haematuria** | 50 | 68 | 118 |
| **Total** | 101 | 95 | 196 |

**Supplemental table S2: 2x2 table for elevated IgM RF and definite endocarditis**

|  | **Endocarditis** | **No endocarditis** | **Total** |
| --- | --- | --- | --- |
| **IgM RF** | 13 | 9 | 22 |
| **No IgM RF** | 54 | 50 | 104 |
| **Total** | 67 | 59 | 126 |

**Supplemental table S3: 2x2 tables for Roth’s Spots and definite endocarditis**

|  | Endocarditis | No endocarditis | Total |
| --- | --- | --- | --- |
| **Roth’s spots** | 5 | 1 | 6 |
| **No Roth’s spots** | 57 | 57 | 114 |
| **Total** | 62 | 58 | 120 |
